# Crystal structure of 2,3,5,6-tetra­kis­(pyridin-2-yl)pyrazine hydrogen peroxide 4.75-solvate

**DOI:** 10.1107/S2056989017015328

**Published:** 2017-10-31

**Authors:** Mger A. Navasardyan, Stanislav I. Bezzubov, Lyudmila G. Kuz’mina, Petr V. Prikhodchenko, Andrei V. Churakov

**Affiliations:** a Institute of General and Inorganic Chemistry, Russian Academy of Sciences, Leninskii prosp. 31, Moscow 119991, Russian Federation

**Keywords:** peroxosolvate, disorder, H-bonded chain, crystal structure

## Abstract

The structure of the title co-crystal, C_24_H_16_N_6_·4.75H_2_O_2_, consists of a 2,3,5,6-tetra­kis­(pyridin-2-yl)pyrazine coformer and hydrogen peroxide solvent mol­ecules in an overall ratio of 1:4.75. Three of the six H_2_O_2_ mol­ecules modelled in the structure were found to be cross-orientationally disordered over two positions with occupancy ratios 0.846 (9):0.154 (9), 0.75 (2):0.25 (2), and 0.891 (9):0.109 (9). In the crystal, all of the peroxide mol­ecules are linked into hydrogen-bonded chains that propagate parallel to the *a* axis. These chains are further linked by O—H⋯N hydrogen bonds to the pyridine groups of the main mol­ecule.

## Abstract 2

The structure of title co-crystal consists of a 2,3,5,6-tetra­kis­(pyridin-2-yl)pyrazine coformer and hydrogen peroxide solvent mol­ecules in a ratio of 1:4.75.

## Chemical context   

Peroxosolvates are solids that contain H_2_O_2_ mol­ecules in a manner analogous to the water in crystalline hydrates. Nowadays, some peroxosolvates find widespread use as environmentally friendly decontaminating and bleaching compounds (Jakob *et al.*, 2012 ▸), and as oxidizing agents in organic synthesis (Ahn *et al.*, 2015 ▸). Hydrogen bonding in peroxosolvates is of particular inter­est because it may be used for modelling of hydrogen peroxide behaviour in various significant biochemical processes, especially oxidative stress and transport through cellular membranes (Kapustin *et al.*, 2014 ▸).
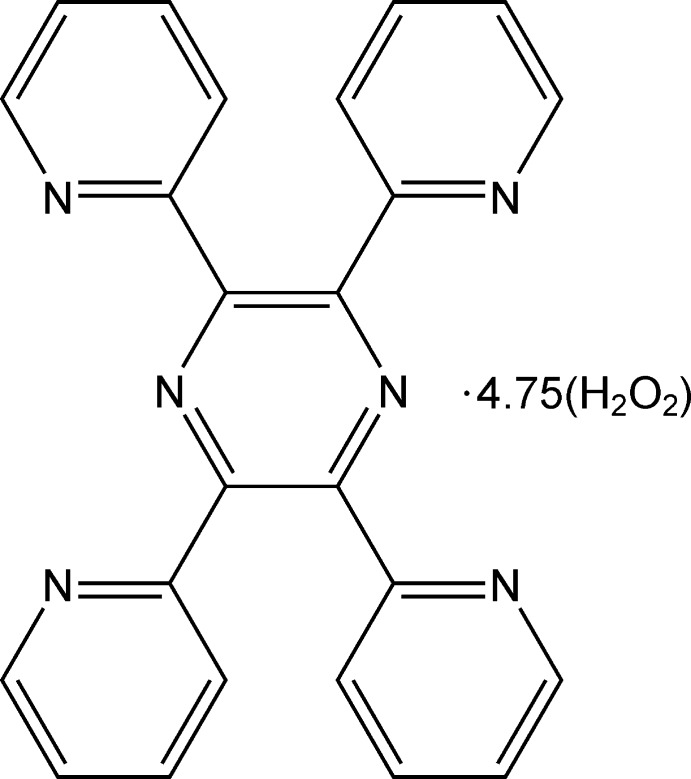


## Structural commentary   

**Figure 1:**
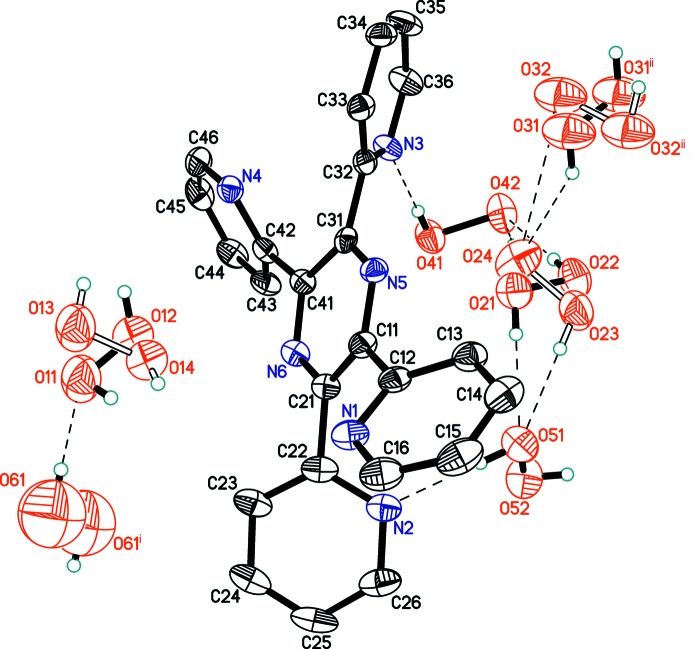
Labelling scheme for organic coformer and six crystallographically independent peroxide mol­ecules. Displacement ellipsoids are shown at the 50% probability level. Hydrogen bonds are drawn as dashed lines. [Symmetry codes: (i) 1 − *x*, −*y*, 1 − *z*; (ii) −*x*, 2 − *y*, 1 − *z*.]

**Figure 2:**
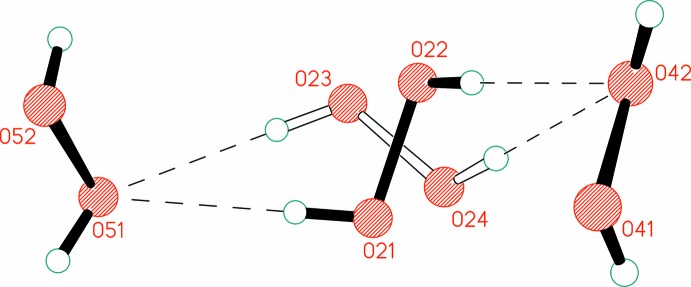
Mol­ecule Per2 cross-orientationally disordered over two positions, showing hydrogen bonds (drawn as dashed lines) to mol­ecules Per4 and Per5. The minor component of disorder is depicted with open bonds.

The title structure consists of a 2,3,5,6-tetra­kis­(pyridin-2-yl)pyrazine coformer and six crystallographically independent peroxide mol­ecules (Fig. 1 ▸), namely Per1 (major occupancy component H11/O11/O12/H12, minor component H13/O13/O14/H14); Per2 (major occupancy component H21/O21/O22/H22, minor component H23/O23/O24/H24); Per3 (major occupancy component H31/O31/O31/H31, minor component H32/O32/O32/H32); Per4 (H41/O41/O42/H42); Per5 (H51/O51/O52/H52); Per6 (H61/O61/O61/H61). Mol­ecules Per1, Per2, Per4, Per5 occupy general positions and thus exhibit a skew geometry. Mol­ecules Per3 and Per6 lie on inversion centres. Three of the six H_2_O_2_ mol­ecules are cross-orientationally disordered over two positions (Fig. 2 ▸). This type of disorder was previously reported for several inorganic peroxosolvates (Adams & Pritchard, 1977 ▸; Carrondo *et al.*, 1977 ▸; Pritchard & Islam, 2003 ▸; Medvedev *et al.*, 2012 ▸).

**Figure 3:**
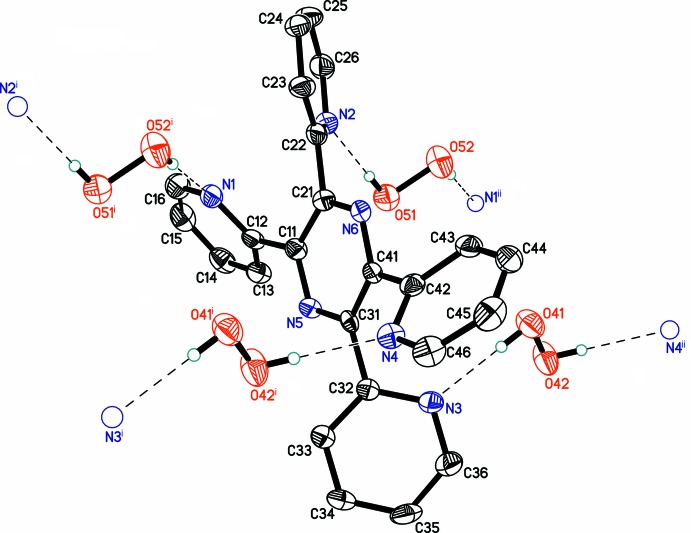
Organic coformer with hydrogen-bonded peroxide mol­ecules. Displacement ellipsoids are shown at the 50% probability level. Hydrogen bonds are drawn as dashed lines. [Symmetry codes: (i) *x*, −1 + *y*, *z*; (ii) *x*, 1 + *y*, *z*.]

In the organic mol­ecule, all four pyridin-2-yl substituents are significantly inclined with respect to the central pyrazine ring (Fig. 3 ▸), such that the N—C—C—N torsion angles range between 130.8 (6) and 140.0 (4)°. Similar conformations have been observed for all three known polymorphs of the pure coformer (Bock *et al.*, 1992 ▸; Behrens & Rehder, 2009 ▸; Malecki, 2010 ▸). Of structural significance, the pairs of pyridinyl nitro­gen atoms N1, N4 and N2, N3 are located at opposite sides of the central pyrazine ring. This arrangement clearly facilitates the organization of hydrogen-bonded chains in the structure (see below). All four pyridinyl nitro­gen atoms are involved as hydrogen-bond acceptors, but neither of the pyrazine N atoms participate in hydrogen bonding, presumably because of steric hindrance.

In the peroxide mol­ecules, the O—O distances range between 1.44 (4) and 1.485 (5) Å. The mean value of 1.465 Å is close to those previously observed in the accurately determined structures of crystalline hydrogen peroxide [1.461 (3) Å; Savariault & Lehmann, 1980 ▸] and urea perhydrate [1.4573 (8) Å; Fritchie & McMullan, 1981 ▸].

**Figure 4:**
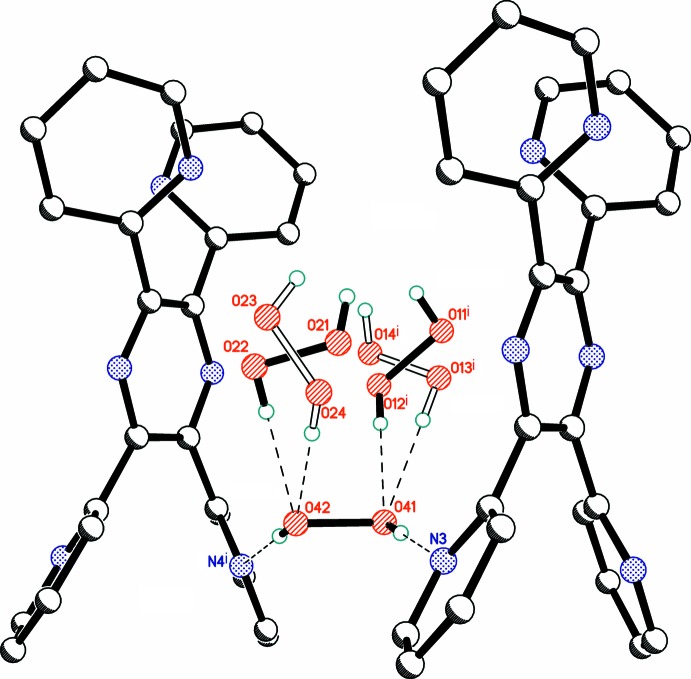
Hydrogen bonds formed by mol­ecule Per4. Minor components of disorder are drawn with open bonds. Hydrogen bonds are drawn as dashed lines. [Symmetry code: (i) *x*, 1 + *y*, *z*.]

The ordered mol­ecules Per4 and Per5 form four hydrogen bonds (two as donor and two as acceptor) in [2,2] mode (Fig. 4 ▸). This coordination environment of the peroxide mol­ecules is the most common arrangement in organic peroxosolvates (Prikhodchenko *et al.*, 2011 ▸). In contrast, the disordered or partially occupied mol­ecules Per1, Per2, Per3, and Per6 are involved in just two or three hydrogen bonds with adjacent peroxide mol­ecules, but not with the organic coformer. It should be noted that the maximum number of hydrogen bonds possible for H_2_O_2_ is six (two as donor and four as acceptor), but such cases are quite rare (Chernyshov *et al.*, 2017 ▸).

## Supra­molecular features   

**Figure 5:**
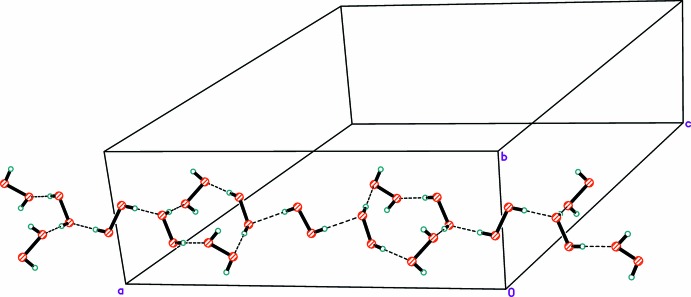
Peroxide hydrogen-bonded chains parallel to the *a*-axis. Minor components of disorder are not shown for clarity. Hydrogen bonds are drawn as dashed lines.

In the crystal, all six peroxide mol­ecules are linked into hydrogen-bonded chains that propagate parallel to the *a*-axis (Table 1 ▸, Fig. 5 ▸). To the best of our knowledge, this is only the second example of hydrogen-bonded chains formed exclusively from peroxide mol­ecules. Recently we reported the structure of thymine peroxosolvate obtained from 98% hydrogen peroxide (Chernyshov *et al.*, 2017 ▸). However, in the latter compound, the peroxide chains are very simple (see Scheme below), belonging to the C1 type according to the Infantes–Motherwell notation of water clusters (Infantes & Motherwell, 2002 ▸). In the title structure, the chains represent the more complicated *T*4(0)*A*1 motif (Fig. 5 ▸).
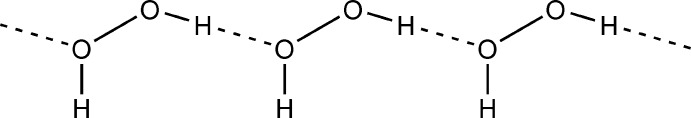


The peroxide chains are inter­connected *via* the organic mol­ecules by moderate HOO—H⋯N hydrogen bonds. Despite the aromatic nature of organic coformer, no π–π stacking or T-shaped C—H⋯π inter­molecular inter­actions are observed in the structure. Thus, hydrogen bonding plays the predominant role in the crystal packing.

## Database survey   

The Cambridge Structural Database (Version 5.38: Groom *et al.*, 2016 ▸) contains data for 72 individual ‘true’ peroxosolvates (78 refcodes), in which the peroxide mol­ecules do not form direct bonds to metal atoms. A few of these represent examples of mixed halogen–peroxide chains of general formula ⋯Hal^−^⋯(H_2_O_2_)_*n*_⋯Hal^−^⋯(H_2_O_2_)_k_⋯ (*n*, *k* = 1, 2; Hal = Cl, Br; CAZHAN, CAZHER, CAZHIV, CAZHOB, CAZHUH, CAZJAP: Churakov *et al.*, 2005 ▸), mixed carbonate–peroxide chains (WUXSIT: Medvedev *et al.*, 2012 ▸) and disordered mixed peroxide–water chains (WINSAO: Churakov & Howard, 2007 ▸; QOHXUH: Laus *et al.*, 2008 ▸).

## Synthesis and crystallization   

98% Hydrogen peroxide was prepared by an extraction method from serine peroxosolvate (Wolanov *et al.*, 2010 ▸). Colourless prismatic crystals of the title compound were obtained by cooling a saturated solution (r.t.) of 2,3,5,6-tetra­kis­(pyridin-2-yl)pyrazine (Aldrich) in 96% hydrogen peroxide to 255 K.

Several crystals were examined. All of them exhibited poor crystallinity, presumably as a result of the rather extensive disorder of the peroxide mol­ecules.

Handling procedures for concentrated hydrogen peroxide have been described in detail (danger of explosion!) by Schumb *et al.* (1955 ▸).

## Refinement   

Crystal data, data collection and structure refinement details are summarized in Table 2 ▸. Three of the six H_2_O_2_ mol­ecules were found to be cross-orientationally disordered over two positions with occupancy ratios 0.846 (9):0.154 (9), 0.75 (2):0.25 (2), and 0.891 (9):0.109 (9), and were refined with restrained O—O distances.

The centrosymmetric peroxide mol­ecule modelled as H61/O61/O61^i^/H61^i^ [symmetry code: (i) 1 − *x*, −*y*, 1 − *z*] was found to be partially occupied. Simultaneous refinement of occupancy and thermal parameters for atom O61 was not stable and resulted in oscillating occupancies between 0.46 and 0.53 for consecutive cycles of refinement. It was therefore fixed at 0.5 for the final refinement.

Aromatic H atoms were placed in calculated positions with C—H = 0.95 Å and refined as riding atoms with relative isotropic displacement parameters *U*_iso_(H) = 1.2*U*_eq_(C). Peroxide hydrogen atoms were placed on the lines connecting hydrogen-bonded atoms at a distance of 0.80 Å from the corresponding O atoms. They were refined as riding atoms with relative isotropic displacement parameters *U*_iso_(H) = 1.5*U*_eq_(O).

## Supplementary Material

Crystal structure: contains datablock(s) I. DOI: 10.1107/S2056989017015328/pk2607sup1.cif

Structure factors: contains datablock(s) I. DOI: 10.1107/S2056989017015328/pk2607Isup2.hkl

Supporting information file. DOI: 10.1107/S2056989017015328/pk2607Isup3.cml

CCDC reference: 1581165

Additional supporting information:  crystallographic information; 3D view; checkCIF report

## Figures and Tables

**Table 1:** Hydrogen-bond geometry (Å, °)

*D*—H⋯*A*	*D*—H	H⋯*A*	*D*⋯*A*	*D*—H⋯*A*
O11—H11⋯O52^i^	0.80	1.99	2.793 (7)	179
O12—H12⋯O41^i^	0.80	1.99	2.789 (7)	180
O13—H13⋯O41^i^	0.80	1.89	2.69 (4)	180
O14—H14⋯O52^i^	0.80	2.05	2.85 (3)	179
O21—H21⋯O51	0.80	2.02	2.820 (9)	180
O22—H22⋯O42	0.80	2.07	2.866 (11)	180
O23—H23⋯O51	0.80	1.96	2.77 (3)	180
O24—H24⋯O42	0.80	1.84	2.64 (3)	180
O31—H31⋯O22	0.80	2.15	2.947 (9)	176
O32—H32⋯O24	0.80	2.34	3.14 (5)	173
O41—H41⋯N3	0.80	1.93	2.729 (5)	180
O42—H42⋯N4^ii^	0.80	1.97	2.770 (5)	180
O51—H51⋯N2	0.80	1.94	2.737 (5)	180
O52—H52⋯N1^ii^	0.80	1.94	2.740 (5)	180
O61—H61⋯O11	0.80	1.64	2.440 (17)	178

Symmetry codes: (i) 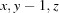; (ii) 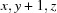.

**Table 2:** Experimental details

Crystal data	
Chemical formula	C_24_H_16_N_6_·4.75H_2_O_2_
*M*_r_	550.00
Crystal system, space group	Monoclinic, *P*2_1_/*n*
Temperature (K)	150
*a*, *b*, *c* (Å)	19.000 (7), 7.382 (3), 20.212 (7)
β (°)	114.271 (5)
*V* (Å^3^)	2584.3 (16)
*Z*	4
Radiation type	Mo *K*α
μ (mm^−1^)	0.11
Crystal size (mm)	0.40 × 0.40 × 0.30

Data collection	
Diffractometer	Bruker SMART APEXII
Absorption correction	Multi-scan (*SADABS*; Bruker, 2008 ▸)
*T*_min_, *T*_max_	0.957, 0.967
No. of measured, independent and observed [*I* > 2σ(*I*)] reflections	16397, 4563, 3870
*R*_int_	0.035
(sin θ/λ)_max_ (Å^−1^)	0.596

Refinement	
*R*[*F* ^2^ > 2σ(*F* ^2^)], *wR*(*F* ^2^), *S*	0.090, 0.191, 1.17
No. of reflections	4563
No. of parameters	388
No. of restraints	3
H-atom treatment	H-atom parameters constrained
Δρ_max_, Δρ_min_ (e Å^−3^)	0.39, −0.36

Computer programs: *APEX2* and *SAINT* (Bruker, 2008 ▸) and *SHELXTL* (Sheldrick, 2008 ▸).

## supplementary crystallographic information

### Crystal data

**Table:**

C_24_H_16_N_6_·4.75H_2_O_2_	*F*(000) = 1150
*M**_r_* = 550.00	*D*_x_ = 1.414 Mg m^−^^3^
Monoclinic, *P*2_1_/*n*	Mo *K*α radiation, λ = 0.71073 Å
Hall symbol: -P 2yn	Cell parameters from 5939 reflections
*a* = 19.000 (7) Å	θ = 2.2–30.1°
*b* = 7.382 (3) Å	µ = 0.11 mm^−^^1^
*c* = 20.212 (7) Å	*T* = 150 K
β = 114.271 (5)°	Prism, colourless
*V* = 2584.3 (16) Å^3^	0.40 × 0.40 × 0.30 mm
*Z* = 4	

### Data collection

**Table:**

Bruker SMART APEXII diffractometer	4563 independent reflections
Radiation source: fine-focus sealed tube	3870 reflections with *I* > 2σ(*I*)
Graphite monochromator	*R*_int_ = 0.035
ω scans	θ_max_ = 25.1°, θ_min_ = 1.2°
Absorption correction: multi-scan (SADABS; Bruker, 2008)	*h* = −22→22
*T*_min_ = 0.957, *T*_max_ = 0.967	*k* = −8→8
16397 measured reflections	*l* = −24→23

### Refinement

**Table:**

Refinement on *F*^2^	Secondary atom site location: difference Fourier map
Least-squares matrix: full	Hydrogen site location: inferred from neighbouring sites
*R*[*F*^2^ > 2σ(*F*^2^)] = 0.090	H-atom parameters constrained
*wR*(*F*^2^) = 0.191	*w* = 1/[σ^2^(*F*_o_^2^) + (0.010*P*)^2^ + 10.*P*] where *P* = (*F*_o_^2^ + 2*F*_c_^2^)/3
*S* = 1.17	(Δ/σ)_max_ < 0.001
4563 reflections	Δρ_max_ = 0.39 e Å^−^^3^
388 parameters	Δρ_min_ = −0.36 e Å^−^^3^
3 restraints	Extinction correction: SHELXTL (Sheldrick, 2008), Fc^*^=kFc[1+0.001xFc^2^λ^3^/sin(2θ)]^-1/4^
Primary atom site location: structure-invariant direct methods	Extinction coefficient: 0.0017 (4)

### Special details

**Table:**

Geometry. All esds (except the esd in the dihedral angle between two l.s. planes) are estimated using the full covariance matrix. The cell esds are taken into account individually in the estimation of esds in distances, angles and torsion angles; correlations between esds in cell parameters are only used when they are defined by crystal symmetry. An approximate (isotropic) treatment of cell esds is used for estimating esds involving l.s. planes.
Refinement. Refinement of F^2^ against ALL reflections. The weighted R-factor wR and goodness of fit S are based on F^2^, conventional R-factors R are based on F, with F set to zero for negative F^2^. The threshold expression of F^2^ > 2sigma(F^2^) is used only for calculating R-factors(gt) etc. and is not relevant to the choice of reflections for refinement. R-factors based on F^2^ are statistically about twice as large as those based on F, and R- factors based on ALL data will be even larger.

### Fractional atomic coordinates and isotropic or equivalent isotropic displacement parameters (Å^2^)

**Table:**

	*x*	*y*	*z*	*U*_iso_*/*U*_eq_	Occ. (<1)
O11	0.3518 (4)	−0.0660 (7)	0.4736 (3)	0.0662 (16)	0.846 (9)
H11	0.3640	−0.0353	0.5148	0.099*	0.846 (9)
O12	0.3037 (3)	0.0865 (7)	0.4355 (3)	0.0500 (13)	0.846 (9)
H12	0.2602	0.0537	0.4129	0.075*	0.846 (9)
O13	0.302 (2)	−0.084 (3)	0.4427 (16)	0.0662 (16)	0.154 (9)
H13	0.2574	−0.0674	0.4170	0.099*	0.154 (9)
O14	0.325 (2)	0.101 (4)	0.4650 (18)	0.0500 (13)	0.154 (9)
H14	0.3448	0.0846	0.5079	0.075*	0.154 (9)
O21	0.1982 (4)	0.8976 (8)	0.5494 (5)	0.046 (2)	0.75 (2)
H21	0.2428	0.9008	0.5768	0.068*	0.75 (2)
O22	0.1750 (5)	1.0887 (9)	0.5388 (5)	0.045 (2)	0.75 (2)
H22	0.1578	1.0953	0.4955	0.068*	0.75 (2)
O23	0.2088 (15)	1.053 (3)	0.5761 (17)	0.054 (6)	0.25 (2)
H23	0.2513	1.0114	0.5961	0.081*	0.25 (2)
O24	0.1651 (17)	0.951 (3)	0.5117 (15)	0.056 (7)	0.25 (2)
H24	0.1496	0.9992	0.4729	0.084*	0.25 (2)
O31	0.0252 (3)	0.9210 (7)	0.5133 (3)	0.0661 (17)	0.891 (9)
H31	0.0661	0.9677	0.5224	0.099*	0.891 (9)
O32	−0.014 (2)	0.952 (6)	0.4653 (14)	0.0661 (17)	0.109 (9)
H32	0.0309	0.9595	0.4745	0.099*	0.109 (9)
O41	0.15205 (18)	0.9727 (4)	0.35656 (18)	0.0412 (8)	
H41	0.1267	0.8912	0.3612	0.062*	
O42	0.1137 (2)	1.1114 (4)	0.38369 (18)	0.0440 (9)	
H42	0.1200	1.1955	0.3618	0.066*	
O51	0.35556 (18)	0.9097 (5)	0.64562 (18)	0.0438 (8)	
H51	0.3802	0.8260	0.6415	0.066*	
O52	0.3959 (2)	1.0412 (5)	0.61765 (18)	0.0496 (9)	
H52	0.3851	1.1264	0.6362	0.074*	
O61	0.4922 (9)	−0.0768 (15)	0.5192 (8)	0.149 (6)*	0.50
H61	0.4462	−0.0750	0.5046	0.224*	0.50
N1	0.35882 (19)	0.3326 (5)	0.68106 (18)	0.0299 (8)	
N2	0.43995 (19)	0.6236 (5)	0.63149 (18)	0.0310 (8)	
N3	0.06552 (18)	0.6947 (5)	0.37238 (18)	0.0276 (8)	
N4	0.13565 (19)	0.4025 (5)	0.30774 (17)	0.0270 (8)	
N5	0.19869 (18)	0.5011 (5)	0.53122 (17)	0.0232 (7)	
N6	0.29935 (18)	0.5095 (5)	0.46312 (17)	0.0243 (7)	
C11	0.2751 (2)	0.4834 (6)	0.5698 (2)	0.0242 (9)	
C12	0.3013 (2)	0.4523 (6)	0.6494 (2)	0.0255 (9)	
C13	0.2670 (2)	0.5469 (6)	0.6880 (2)	0.0307 (10)	
H13A	0.2254	0.6277	0.6639	0.037*	
C14	0.2949 (3)	0.5205 (7)	0.7624 (2)	0.0374 (11)	
H14A	0.2723	0.5826	0.7900	0.045*	
C15	0.3561 (3)	0.4026 (7)	0.7959 (2)	0.0414 (12)	
H15A	0.3772	0.3847	0.8470	0.050*	
C16	0.3856 (3)	0.3116 (7)	0.7531 (2)	0.0375 (11)	
H16A	0.4271	0.2296	0.7761	0.045*	
C21	0.3263 (2)	0.4994 (6)	0.5354 (2)	0.0248 (9)	
C22	0.4120 (2)	0.5050 (6)	0.5756 (2)	0.0287 (9)	
C23	0.4586 (2)	0.3985 (7)	0.5539 (2)	0.0329 (10)	
H23A	0.4367	0.3177	0.5140	0.039*	
C24	0.5380 (2)	0.4123 (7)	0.5916 (2)	0.0380 (11)	
H24A	0.5716	0.3398	0.5784	0.046*	
C25	0.5674 (2)	0.5328 (8)	0.6485 (3)	0.0444 (13)	
H25A	0.6217	0.5459	0.6747	0.053*	
C26	0.5169 (2)	0.6350 (7)	0.6671 (3)	0.0396 (11)	
H26A	0.5378	0.7166	0.7068	0.048*	
C31	0.1721 (2)	0.5232 (5)	0.45945 (19)	0.0199 (8)	
C32	0.0876 (2)	0.5562 (5)	0.42050 (19)	0.0215 (8)	
C33	0.0354 (2)	0.4529 (6)	0.4356 (2)	0.0240 (9)	
H33A	0.0530	0.3572	0.4700	0.029*	
C34	−0.0424 (2)	0.4894 (6)	0.4006 (2)	0.0283 (9)	
H34A	−0.0791	0.4196	0.4102	0.034*	
C35	−0.0658 (2)	0.6309 (7)	0.3507 (2)	0.0371 (11)	
H35A	−0.1190	0.6598	0.3256	0.045*	
C36	−0.0101 (2)	0.7290 (7)	0.3384 (2)	0.0356 (11)	
H36A	−0.0265	0.8252	0.3042	0.043*	
C41	0.2231 (2)	0.5160 (5)	0.4245 (2)	0.0217 (8)	
C42	0.1960 (2)	0.5127 (6)	0.3444 (2)	0.0249 (9)	
C43	0.2327 (2)	0.6153 (6)	0.3100 (2)	0.0294 (9)	
H43A	0.2757	0.6893	0.3376	0.035*	
C44	0.2056 (2)	0.6080 (7)	0.2353 (2)	0.0334 (10)	
H44A	0.2299	0.6763	0.2107	0.040*	
C45	0.1427 (3)	0.4993 (7)	0.1970 (2)	0.0372 (11)	
H45A	0.1225	0.4932	0.1456	0.045*	
C46	0.1094 (3)	0.3989 (6)	0.2350 (2)	0.0329 (10)	
H46A	0.0662	0.3245	0.2083	0.039*	

### Atomic displacement parameters (Å^2^)

**Table:**

	*U*^11^	*U*^22^	*U*^33^	*U*^12^	*U*^13^	*U*^23^
O11	0.075 (4)	0.052 (3)	0.057 (3)	0.014 (3)	0.013 (3)	−0.001 (2)
O12	0.053 (3)	0.051 (3)	0.046 (3)	−0.002 (2)	0.020 (3)	0.005 (3)
O13	0.075 (4)	0.052 (3)	0.057 (3)	0.014 (3)	0.013 (3)	−0.001 (2)
O14	0.053 (3)	0.051 (3)	0.046 (3)	−0.002 (2)	0.020 (3)	0.005 (3)
O21	0.045 (3)	0.022 (3)	0.062 (5)	0.006 (2)	0.015 (3)	0.009 (3)
O22	0.054 (5)	0.034 (4)	0.051 (4)	0.011 (3)	0.026 (4)	0.008 (3)
O23	0.053 (12)	0.050 (12)	0.054 (14)	0.018 (9)	0.017 (11)	−0.002 (10)
O24	0.067 (15)	0.039 (12)	0.054 (12)	−0.013 (10)	0.015 (12)	0.015 (10)
O31	0.046 (3)	0.066 (4)	0.084 (4)	−0.006 (2)	0.023 (3)	0.019 (3)
O32	0.046 (3)	0.066 (4)	0.084 (4)	−0.006 (2)	0.023 (3)	0.019 (3)
O41	0.0441 (19)	0.0314 (18)	0.060 (2)	−0.0005 (15)	0.0332 (17)	0.0008 (16)
O42	0.062 (2)	0.0319 (19)	0.055 (2)	0.0017 (16)	0.0406 (18)	0.0050 (16)
O51	0.0428 (19)	0.040 (2)	0.053 (2)	0.0024 (16)	0.0236 (16)	0.0053 (16)
O52	0.062 (2)	0.044 (2)	0.050 (2)	−0.0047 (18)	0.0307 (18)	0.0026 (17)
N1	0.0267 (18)	0.034 (2)	0.0260 (18)	−0.0024 (16)	0.0076 (14)	0.0065 (15)
N2	0.0246 (18)	0.033 (2)	0.0324 (19)	−0.0004 (15)	0.0087 (15)	0.0069 (16)
N3	0.0224 (17)	0.032 (2)	0.0316 (18)	0.0030 (15)	0.0143 (14)	0.0028 (15)
N4	0.0268 (18)	0.031 (2)	0.0245 (17)	0.0013 (15)	0.0114 (14)	−0.0026 (15)
N5	0.0201 (16)	0.0252 (18)	0.0246 (17)	−0.0034 (14)	0.0096 (13)	−0.0016 (14)
N6	0.0235 (17)	0.0241 (18)	0.0271 (17)	0.0006 (14)	0.0123 (14)	0.0058 (14)
C11	0.0234 (19)	0.024 (2)	0.0248 (19)	−0.0030 (16)	0.0097 (16)	−0.0010 (16)
C12	0.0208 (19)	0.031 (2)	0.025 (2)	−0.0072 (17)	0.0096 (16)	−0.0005 (17)
C13	0.026 (2)	0.039 (3)	0.027 (2)	−0.0044 (19)	0.0112 (17)	−0.0007 (18)
C14	0.039 (2)	0.046 (3)	0.031 (2)	−0.012 (2)	0.017 (2)	−0.003 (2)
C15	0.043 (3)	0.050 (3)	0.024 (2)	−0.013 (2)	0.007 (2)	0.004 (2)
C16	0.033 (2)	0.045 (3)	0.029 (2)	−0.003 (2)	0.0070 (19)	0.008 (2)
C21	0.0216 (19)	0.026 (2)	0.025 (2)	0.0003 (17)	0.0082 (16)	0.0015 (17)
C22	0.023 (2)	0.037 (3)	0.024 (2)	−0.0014 (18)	0.0079 (16)	0.0115 (19)
C23	0.024 (2)	0.051 (3)	0.026 (2)	0.006 (2)	0.0129 (17)	0.011 (2)
C24	0.026 (2)	0.051 (3)	0.040 (2)	0.010 (2)	0.017 (2)	0.020 (2)
C25	0.019 (2)	0.063 (4)	0.047 (3)	0.004 (2)	0.009 (2)	0.019 (3)
C26	0.028 (2)	0.041 (3)	0.039 (2)	−0.009 (2)	0.0039 (19)	0.008 (2)
C31	0.0243 (19)	0.0136 (19)	0.0240 (19)	−0.0047 (15)	0.0121 (16)	−0.0011 (15)
C32	0.0224 (19)	0.024 (2)	0.0203 (18)	−0.0004 (16)	0.0112 (15)	−0.0025 (16)
C33	0.025 (2)	0.029 (2)	0.0189 (18)	−0.0030 (17)	0.0103 (16)	−0.0022 (16)
C34	0.0204 (19)	0.040 (3)	0.026 (2)	−0.0063 (18)	0.0105 (16)	−0.0057 (19)
C35	0.018 (2)	0.054 (3)	0.036 (2)	0.006 (2)	0.0076 (18)	0.002 (2)
C36	0.027 (2)	0.041 (3)	0.039 (2)	0.007 (2)	0.0141 (19)	0.008 (2)
C41	0.0215 (19)	0.019 (2)	0.027 (2)	−0.0008 (16)	0.0120 (16)	0.0035 (16)
C42	0.0221 (19)	0.028 (2)	0.027 (2)	0.0058 (17)	0.0122 (16)	0.0016 (17)
C43	0.022 (2)	0.040 (3)	0.030 (2)	0.0027 (18)	0.0144 (17)	0.0044 (19)
C44	0.033 (2)	0.044 (3)	0.031 (2)	0.006 (2)	0.0208 (19)	0.009 (2)
C45	0.044 (3)	0.049 (3)	0.023 (2)	0.012 (2)	0.0183 (19)	0.001 (2)
C46	0.035 (2)	0.038 (3)	0.024 (2)	0.001 (2)	0.0107 (18)	−0.0081 (19)

### Geometric parameters (Å, º)

**Table:**

O11—H11	0.8000	C12—C13	1.392 (6)
O12—H12	0.8001	C13—C14	1.387 (6)
O13—H13	0.8000	C13—H13A	0.9500
O14—H14	0.7999	C14—C15	1.386 (7)
O21—O22	1.467 (12)	C14—H14A	0.9500
O21—H21	0.8000	C15—C16	1.383 (7)
O22—H22	0.8002	C15—H15A	0.9500
O23—O24	1.44 (4)	C16—H16A	0.9500
O23—H23	0.8001	C21—C22	1.491 (5)
O24—H24	0.7998	C22—C23	1.384 (6)
O31—O31^i^	1.465 (9)	C23—C24	1.386 (6)
O31—H31	0.7999	C23—H23A	0.9500
O31—H32	0.8813	C24—C25	1.378 (7)
O32—O32^i^	1.463 (13)	C24—H24A	0.9500
O32—H32	0.7999	C25—C26	1.389 (7)
O41—O42	1.486 (4)	C25—H25A	0.9500
O41—H41	0.7997	C26—H26A	0.9500
O42—H42	0.8001	C31—C41	1.415 (5)
O51—O52	1.485 (5)	C31—C32	1.489 (5)
O51—H51	0.8002	C32—C33	1.382 (5)
O52—H52	0.8001	C33—C34	1.377 (5)
O61—O61^ii^	1.471 (10)	C33—H33A	0.9500
O61—H61	0.7999	C34—C35	1.391 (6)
N1—C16	1.340 (5)	C34—H34A	0.9500
N1—C12	1.345 (5)	C35—C36	1.387 (6)
N2—C26	1.340 (5)	C35—H35A	0.9500
N2—C22	1.354 (6)	C36—H36A	0.9500
N3—C36	1.338 (5)	C41—C42	1.483 (5)
N3—C32	1.353 (5)	C42—C43	1.394 (6)
N4—C46	1.345 (5)	C43—C44	1.381 (6)
N4—C42	1.352 (5)	C43—H43A	0.9500
N5—C31	1.336 (5)	C44—C45	1.383 (6)
N5—C11	1.342 (5)	C44—H44A	0.9500
N6—C41	1.335 (5)	C45—C46	1.391 (6)
N6—C21	1.337 (5)	C45—H45A	0.9500
C11—C21	1.416 (5)	C46—H46A	0.9500
C11—C12	1.493 (5)		
			
O12—O11—H11	100.6	C22—C23—C24	118.6 (4)
O11—O12—H12	110.1	C22—C23—H23A	120.7
H12—O12—H14	128.2	C24—C23—H23A	120.7
H13—O12—H14	107.7	C25—C24—C23	118.8 (4)
O14—O13—H13	99.5	C25—C24—H24A	120.6
H11—O14—H12	115.4	C23—C24—H24A	120.6
O13—O14—H14	97.8	C24—C25—C26	119.2 (4)
O22—O21—H21	104.0	C24—C25—H25A	120.4
O21—O22—H22	100.4	C26—C25—H25A	120.4
O24—O23—H23	110.2	N2—C26—C25	122.9 (5)
O23—O24—H24	119.8	N2—C26—H26A	118.5
O31^i^—O31—H31	99.7	C25—C26—H26A	118.5
O32^i^—O32—H32	78.8	N5—C31—C41	120.5 (3)
O42—O41—H41	93.7	N5—C31—C32	116.0 (3)
O41—O42—H42	97.0	C41—C31—C32	123.5 (3)
O52—O51—H51	92.9	N3—C32—C33	122.5 (4)
O51—O52—H52	93.7	N3—C32—C31	116.9 (3)
O61^ii^—O61—H61	102.7	C33—C32—C31	120.6 (3)
C16—N1—C12	117.7 (4)	C34—C33—C32	119.6 (4)
C26—N2—C22	117.1 (4)	C34—C33—H33A	120.2
C36—N3—C32	117.5 (4)	C32—C33—H33A	120.2
C46—N4—C42	117.5 (4)	C33—C34—C35	118.4 (4)
C31—N5—C11	118.6 (3)	C33—C34—H34A	120.8
C41—N6—C21	118.5 (3)	C35—C34—H34A	120.8
N5—C11—C21	120.2 (3)	C36—C35—C34	118.8 (4)
N5—C11—C12	116.4 (3)	C36—C35—H35A	120.6
C21—C11—C12	123.4 (3)	C34—C35—H35A	120.6
N1—C12—C13	122.6 (4)	N3—C36—C35	123.2 (4)
N1—C12—C11	117.4 (4)	N3—C36—H36A	118.4
C13—C12—C11	120.0 (4)	C35—C36—H36A	118.4
C14—C13—C12	118.6 (4)	N6—C41—C31	120.7 (3)
C14—C13—H13A	120.7	N6—C41—C42	116.3 (3)
C12—C13—H13A	120.7	C31—C41—C42	122.9 (3)
C15—C14—C13	119.2 (4)	N4—C42—C43	122.6 (4)
C15—C14—H14A	120.4	N4—C42—C41	116.4 (3)
C13—C14—H14A	120.4	C43—C42—C41	121.0 (4)
C16—C15—C14	118.3 (4)	C44—C43—C42	119.1 (4)
C16—C15—H15A	120.9	C44—C43—H43A	120.5
C14—C15—H15A	120.9	C42—C43—H43A	120.5
N1—C16—C15	123.6 (4)	C43—C44—C45	118.8 (4)
N1—C16—H16A	118.2	C43—C44—H44A	120.6
C15—C16—H16A	118.2	C45—C44—H44A	120.6
N6—C21—C11	120.6 (3)	C44—C45—C46	119.0 (4)
N6—C21—C22	115.8 (3)	C44—C45—H45A	120.5
C11—C21—C22	123.5 (3)	C46—C45—H45A	120.5
N2—C22—C23	123.3 (4)	N4—C46—C45	123.0 (4)
N2—C22—C21	116.0 (4)	N4—C46—H46A	118.5
C23—C22—C21	120.6 (4)	C45—C46—H46A	118.5
			
C31—N5—C11—C21	3.9 (6)	C41—C31—C32—N3	−47.1 (5)
C31—N5—C11—C12	−177.8 (4)	N5—C31—C32—C33	−45.2 (5)
C16—N1—C12—C13	−3.0 (6)	C41—C31—C32—C33	135.2 (4)
C16—N1—C12—C11	176.3 (4)	N3—C32—C33—C34	0.4 (6)
N5—C11—C12—N1	140.0 (4)	C31—C32—C33—C34	178.0 (3)
C21—C11—C12—N1	−41.8 (6)	C32—C33—C34—C35	−0.1 (6)
N5—C11—C12—C13	−40.7 (6)	C33—C34—C35—C36	0.0 (6)
C21—C11—C12—C13	137.6 (4)	C32—N3—C36—C35	0.5 (7)
N1—C12—C13—C14	2.0 (6)	C34—C35—C36—N3	−0.2 (7)
C11—C12—C13—C14	−177.3 (4)	C21—N6—C41—C31	3.8 (6)
C12—C13—C14—C15	0.5 (7)	C21—N6—C41—C42	−175.4 (4)
C13—C14—C15—C16	−1.8 (7)	N5—C31—C41—N6	−8.3 (6)
C12—N1—C16—C15	1.6 (7)	C32—C31—C41—N6	171.2 (4)
C14—C15—C16—N1	0.7 (7)	N5—C31—C41—C42	170.9 (4)
C41—N6—C21—C11	4.3 (6)	C32—C31—C41—C42	−9.6 (6)
C41—N6—C21—C22	−176.0 (4)	C46—N4—C42—C43	−2.3 (6)
N5—C11—C21—N6	−8.4 (6)	C46—N4—C42—C41	179.2 (4)
C12—C11—C21—N6	173.4 (4)	N6—C41—C42—N4	135.6 (4)
N5—C11—C21—C22	171.9 (4)	C31—C41—C42—N4	−43.6 (5)
C12—C11—C21—C22	−6.2 (7)	N6—C41—C42—C43	−42.8 (6)
C26—N2—C22—C23	−0.4 (6)	C31—C41—C42—C43	137.9 (4)
C26—N2—C22—C21	−178.1 (4)	N4—C42—C43—C44	1.5 (6)
N6—C21—C22—N2	130.8 (4)	C41—C42—C43—C44	179.9 (4)
C11—C21—C22—N2	−49.6 (6)	C42—C43—C44—C45	0.2 (6)
N6—C21—C22—C23	−47.0 (6)	C43—C44—C45—C46	−1.0 (7)
C11—C21—C22—C23	132.7 (4)	C42—N4—C46—C45	1.6 (6)
N2—C22—C23—C24	0.6 (6)	C44—C45—C46—N4	0.1 (7)
C21—C22—C23—C24	178.1 (4)	H11—O11—O12—H12	−116.1
C22—C23—C24—C25	−0.8 (6)	H13—O13—O14—H14	127.8
C23—C24—C25—C26	0.9 (7)	H21—O21—O22—H22	125.3
C22—N2—C26—C25	0.5 (6)	H23—O23—O24—H24	−118.1
C24—C25—C26—N2	−0.8 (7)	H31—O31—O31^i^—H31^i^	180.0
C11—N5—C31—C41	4.1 (6)	H32—O32—O32^i^—H32^i^	180.0
C11—N5—C31—C32	−175.5 (4)	H41—O41—O42—H42	−152.1
C36—N3—C32—C33	−0.6 (6)	H51—O51—O52—H52	−158.2
C36—N3—C32—C31	−178.3 (4)	H61—O61—O61^ii^—H61^ii^	180.0
N5—C31—C32—N3	132.5 (4)		

Symmetry codes: (i) −*x*, −*y*+2, −*z*+1; (ii) −*x*+1, −*y*, −*z*+1.

### Hydrogen-bond geometry (Å, º)

**Table:**

*D*—H···*A*	*D*—H	H···*A*	*D*···*A*	*D*—H···*A*
O11—H11···O52^iii^	0.80	1.99	2.793 (7)	179
O12—H12···O41^iii^	0.80	1.99	2.789 (7)	180
O13—H13···O41^iii^	0.80	1.89	2.69 (4)	180
O14—H14···O52^iii^	0.80	2.05	2.85 (3)	179
O21—H21···O51	0.80	2.02	2.820 (9)	180
O22—H22···O42	0.80	2.07	2.866 (11)	180
O23—H23···O51	0.80	1.96	2.77 (3)	180
O24—H24···O42	0.80	1.84	2.64 (3)	180
O31—H31···O22	0.80	2.15	2.947 (9)	176
O32—H32···O24	0.80	2.34	3.14 (5)	173
O41—H41···N3	0.80	1.93	2.729 (5)	180
O42—H42···N4^iv^	0.80	1.97	2.770 (5)	180
O51—H51···N2	0.80	1.94	2.737 (5)	180
O52—H52···N1^iv^	0.80	1.94	2.740 (5)	180
O61—H61···O11	0.80	1.64	2.440 (17)	178

Symmetry codes: (iii) *x*, *y*−1, *z*; (iv) *x*, *y*+1, *z*.

## References

[bb2] Adams, J. M. & Pritchard, R. G. (1977). *Acta Cryst.* B**33**, 3650–3653.

[bb3] Ahn, S. H., Cluff, K. J., Bhuvanesh, N. & Blümel, J. (2015). *Angew. Chem. Int. Ed.* **54**, 13341–13345. 10.1002/anie.201505291 26457679

[bb4] Behrens, A. & Rehder, D. (2009). CCDC 261615: CSD communication.

[bb5] Bock, H., Vaupel, T., Näther, C., Ruppert, K. & Havlas, Z. (1992). *Angew. Chem. Int. Ed. Engl.* **31**, 299–301.

[bb6] Bruker (2008). *APEX2*, *SADABS* and *SAINT*. Bruker AXS Inc., Madison, Wisconsin, USA.

[bb1] Carrondo, M. A. A. F. de C. T., Griffith, W. P., Jones, D. P. & Skapski, A. C. (1977). *J. Chem. Soc. Dalton Trans.* pp. 2323–2327.

[bb7] Chernyshov, I. Yu., Vener, M. V., Prikhodchenko, P. V., Medvedev, A. G., Lev, O. & Churakov, A. V. (2017). *Cryst. Growth Des.* **17**, 214–220.

[bb8] Churakov, A. V. & Howard, J. A. K. (2007). *Acta Cryst.* E**63**, o4483.

[bb9] Churakov, A. V., Prikhodchenko, P. V. & Howard, J. A. K. (2005). *CrystEngComm*, **7**, 664–669.

[bb10] Fritchie, C. J. & McMullan, R. K. (1981). *Acta Cryst.* B**37**, 1086–1091.

[bb11] Groom, C. R., Bruno, I. J., Lightfoot, M. P. & Ward, S. C. (2016). *Acta Cryst.* B**72**, 171–179. 10.1107/S2052520616003954 PMC4822653 27048719

[bb12] Infantes, L. & Motherwell, S. (2002). *CrystEngComm*, **4**, 454–461.

[bb13] Jakob, H., Leininger, S., Lehmann, T., Jacobi, S. & Gutewort, S. (2012). *Ullmann’s Encyclopedia of Industrial Chemistry*, pp 1–33. Weinheim: Wiley-VCH Verlag GmbH & Co. KGaA.

[bb14] Kapustin, E. A., Minkov, V. S. & Boldyreva, E. V. (2014). *CrystEngComm*, **16**, 10165–10168.

[bb15] Laus, G., Kahlenberg, V., Wurst, K., Lörting, T. & Schottenberger, H. (2008). *CrystEngComm*, **10**, 1638–1644.

[bb16] Malecki, J. G. (2010). Private communication (refcode 783938). CCDC, Cambridge, England.

[bb17] Medvedev, A. G., Mikhaylov, A. A., Churakov, A. V., Prikhodchenko, P. V. & Lev, O. (2012). *Acta Cryst.* C**68**, i20–i24. 10.1107/S0108270112006701 22382531

[bb18] Prikhodchenko, P. V., Medvedev, A. G., Tripol’skaya, T. A., Churakov, A. V., Wolanov, Y., Howard, J. A. K. & Lev, O. (2011). *CrystEngComm*, **13**, 2399–2407.

[bb19] Pritchard, R. G. & Islam, E. (2003). *Acta Cryst.* B**59**, 596–605. 10.1107/s0108768103012291 14586079

[bb20] Savariault, J. M. & Lehmann, M. S. (1980). *J. Am. Chem. Soc.* **102**, 1298–1303.

[bb21] Schumb, W. C., Satterfield, C. N. & Wentworth, R. P. (1955). *Hydrogen peroxide*. New York: Reinhold Publishing Corp.

[bb22] Sheldrick, G. M. (2008). *Acta Cryst.* A**64**, 112–122. 10.1107/S0108767307043930 18156677

[bb23] Wolanov, Y., Lev, O., Churakov, A. V., Medvedev, A. G., Novotortsev, V. M. & Prikhodchenko, P. V. (2010). *Tetrahedron*, **66**, 5130–5133.

